# RETRACTION: Comparison of the Effects of Laparoscopic and Open Hysterectomy on Surgical Site Wound Infections in Patients With Endometrial Cancer: A Meta‐Analysis

**DOI:** 10.1111/iwj.70322

**Published:** 2025-03-06

## Abstract

**RETRACTION:** YangN. and ZhouF. , “Comparison of the Effects of Laparoscopic and Open Hysterectomy on Surgical Site Wound Infections in Patients With Endometrial Cancer: A Meta‐Analysis,” International Wound Journal 21, no. 2 (2024): e14415, 10.1111/iwj.14415.PMC1082462637743352

The above article, published online on 24 September 2023, in Wiley Online Library (http://onlinelibrary.wiley.com/), has been retracted by agreement between the journal Editor in Chief, Professor Keith Harding; and John Wiley & Sons Ltd. Following an investigation by the publisher, all parties have concluded that this article was accepted solely on the basis of a compromised peer review process. The editors have therefore decided to retract the article. The authors did not respond to our notice regarding the retraction.

**RETRACTION:** N. Yang and F. Zhou , “Comparison of the Effects of Laparoscopic and Open Hysterectomy on Surgical Site Wound Infections in Patients With Endometrial Cancer: A Meta‐Analysis,” International Wound Journal 21, no. 2 (2024): e14415, 10.1111/iwj.14415.PMC1082462637743352

The above article, published online on 24 September 2023, in Wiley Online Library (http://onlinelibrary.wiley.com/), has been retracted by agreement between the journal Editor in Chief, Professor Keith Harding; and John Wiley & Sons Ltd. Following an investigation by the publisher, all parties have concluded that this article was accepted solely on the basis of a compromised peer review process. The editors have therefore decided to retract the article. The authors did not respond to our notice regarding the retraction.

